# Hospital Progress and Finance

**Published:** 1924-06

**Authors:** 


### HOSPITAL PROGRESS AND FINANCE.

### THE HOSPITAL BEDS INQUIRY.

The recommendations of the Voluntary Hospital Commission upon the extent of the need for more hospital beds and the best means of supplying them will be awaited with interest. The motive for the inquiry was supplied by a question in Parliament, which led the Minister of Health to submit it to the Voluntary Hospitals Commission. To those who have always had to admit that the inadequacy of the number of beds was the chief defect of the voluntary system, it may seem that we have already waited long for such an inquiry to be made. The Ministry of Health has now become firmly established, but, being a Government department, could hardly undertake the inquiry satisfactorily where the voluntary system was concerned. The appointment of the Voluntary Hospitals Commission has provided the proper instrument, and we are glad that it is having this new opportunity of service, now that its immediate work in the distribution of the grant has been accomplished. The future of the voluntary system depends a good deal upon the closer co-ordination that the Commission is able to receive, and its second great task should reveal, not only the extent of the present weakness, but the best common policy for reducing it to more reasonable dimensions. When the conference of representatives of the local hospital committees meets at the Ministry of Health, on June 18, this question will be the chief topic of discussion.

### COMPLETING THE ROCKEFELLER GIFT.

To complete the work started in 1920, when Mr. Rockefeller, senior, gave £1,205,000 to establish in London an advanced school of medical education and research, an appeal for £180,000 is being made. A year ago the King and Queen laid the foundation-stone of the new buildings, and on May 31 the anniversary, a banquet, reception, and ball are to be held at Lansdowne House, lent by Mr. Selfridge. The sum, of which about half is assured, will provide sixty beds in the new obstetric hospital, and representatives of those who have endowed beds will be received by the Duke and Duchess of York and other members of the Royal Family, who will be present at the reception. Sir Ernest Hatch, chairman of University College Hospital, is one of the signatories of the appeal, which will of course realise the most far-reaching hospital scheme that a single donor has make possible in London. The maintenance of these beds was the only act left to public generosity in the matter, and it is doubtful if hospital beds can be put to better use than for the cases contemplated.

### A NEW HOSPITAL FOR SHEFFIELD?

Few legacies have been more timely than that of £40,000 which the late Mr. Robert Halliday has left to the three principal hospitals of Sheffield and the Cherrytree Orphanage. It coincides with a project for a great joint hospital on the outskirts of the city, which the Lord Mayor, Mr. A. J. Blanchard, and Mr. Philip K. Watts, the retiring chairman of the Sheffield Royal Hospital, have respectively advocated and supported. Mr. Watts states that the Joint Hospitals Council is considering the matter, and that the difficulty in the way is the combined debt of the Infirmary and the Hospital, amounting to £80,000. He then went on to announce Mr. Halliday's legacy, which will provide almost half the sum required. To extinguish the debt may prove final, for the penny-in-the-pound scheme is enabling the hospitals to pay their way. If, then, present indebtedness is the "only" difficulty in the way of this ambitious scheme, its undertaking has come suddenly nearer than seemed possible. A chance to clear off the rest of the debt has arisen, and if the scheme is being seriously considered an effort to do this will be the first practical sign.

### A STEP TOWARD COMBINATION.

The Salisbury and District Infirmary League seems to be well on its way to realise the income of £10,000 a year that it was formed to collect for the infirmary. The League has now 20,000 members, and raised last year £3,300. During 1923 the returns from each quarter showed a steady increase, so that it is hoped to see £5,000 raised at the end of the next twelve months. About 20 per cent. of the patients in the infirmary were members of the League, and it is hoped eventually to treble this proportion. The League is also understood to be encouraging co-operation between all the hospitals and nursing associations of the district, and it is even hoped that this will grow into a single organisation in time, to which members will pay a single subscription for the different benefits provided. Mr. Luckham, the president, and Mr. H. T. Rowe, the chairman of the League, have worked hard, and the success of the League has led to many inquiries from other centres anxious to emulate it.

### DISTANT PATIENTS AND WAITING LISTS.

Once more the suggestion is made that the Manchester hospitals are receiving an undue proportion of cases from districts far removed from the boundaries of Manchester and Salford. The reply is that in 1922, the latest analysed year, there were 40,629 such cases, which is roughly 15 per cent. of the whole. That is a considerable proportion, though it is defended on the ground that the patients could not receive the special treatment that they need elsewhere. It may be so, but when it is remembered that the patients come from such places as Bolton, the question arises what, as a matter of fact, are the special treatments available at Manchester which no other hospital in the extended area served can supply. There should be no difficulty in answering this. Since, moreover, the outlying districts do not contribute more than about half the sum which patients coming from them cost, it is worth while for the Manchester hospitals not to receive more distant cases than they alone are equipped to treat. The present long waiting lists weigh heavily upon local sufferers, and a certain discontent is felt in the belief that local beds are needlessly occupied by patients from a distance. The Manchester hospitals need the goodwill of every person in their immediate districts, and a sense of grievance would be removed if it were made plain which special treatments are confined to Manchester, and that patients from towns with good hospitals of their own were admitted when in need of these, or for special reasons in such case. But what if the reasons be that the case is urgent and there is no bed elsewhere ? The scarcity of beds is the real crux, but those we have may be allotted carelessly.

### THE ABERDEEN JOINT SCHEME.

The committee and friends of the Royal Aberdeen Hospital for Sick Children are hoping that the building of a new hospital will be begun shortly. Some £50,000 has been subscribed, of which £4,000 has been spent on the Ashley site. Moreover, the cost of the institution's share of the Joint Hospital site, amounting to £5,000, has been promised by Mr. and Mrs. Herbert Taylor. The Joint Hospital scheme, delayed by difficulties arising from the constitution of the Infirmary, is now relatively straightforward, and by the time of the next annual meeting a start will probably have been made. So ambitious a scheme has necessarily taken time and encountered difficulties, but when it begins to be realised it should prove well worth all that it has cost.

### RESIGNATION AT SHEFFIELD.

Mr. J. W. Robinson has resigned his post as secretary to the Royal Hospital, Sheffield, which he has held for forty-two years. It was in 1882 that Mr. Robinson became secretary to the old Public Hospital and Dispensary, and a great task lay before him in helping to raise the fund of £120,000 for the new hospital buildings, the first wing of which was opened by the King and Queen, then Duke and Duchess of York, in 1895. While the new hospital was being built and the old demolished the work went on, but in those days, of course, the number of patients was much smaller. In 1882 the in-patients amounted to 826 ; last year they were 3,631. The beds have increased from 85 to 316. The years 1908 and 1912 saw further reconstructions, and the work involved by the war and its legacy of difficulties have made Mr. Robinson's period of service arduous as well as long. He has served under five chairmen, and until the Joint Hospitals Council and its successful penny-in-the-pound scheme made maintenance more secure, Sheffield was one of the most difficult centres financially for voluntary hospitals. Mr. Robinson, who feels that a younger man should now shoulder the work, will be greatly missed. His resignation will take effect at midsummer. Following closely upon that of Mr. Philip K. Watts, the chairman, Mr. Robinson's retirement seems to mark the end of a generation whose leaders will feel that their efforts have left the Sheffield hospitals stronger and more secure than they have been for many years. This fact alone is sufficient to show the quality of the work behind them.

### TRADE UNIONS AND HOSPITAL DISCIPLINE.

A curious dispute has occurred between the local trades unions and the Wemyss Memorial Hospital, Buckhaven, Fife. It seems that the nurses, having complained that their morning allowance of tea and bread had been cut off, were dismissed by the matron, Miss Mackay, whereupon the nurses appealed to the trade unions, who resolved that, unless the dismissals were withdrawn, they would suspend their contributions to the hospital. The committee of management is issuing a statement, pending which we refrain from comment. Meantime it is reported that one of the nurses appeared in uniform at a local Labour demonstration and thanked those present for the support they had given to the staff. The meeting passed a resolution pledging itself to withhold all contributions to the hospital until its demands were met, and condemning the hospital secretary for passing on the letter containing these demands to the matron.

### OIL FUEL FOR INSTITUTIONS.

Hospitals have been less prompt than ships in experimenting with or adopting oil fuel, but the latest institution to consider the change is St. Bartholomew's. According to the *Journal*, the idea is to substitute oil for coal in the warming of the building and in the supply of hot water and steam. The belief is that it is not more costly and may prove even more economical, because a more level temperature is obtained, and the expense involved in stoking is reduced. Another advantage is that smoke will be diminished, though the hospital will not deprive itself of the genial coal fires which in winter still add to the pleasantness of the wards, many of which contain old-fashioned open grates. We believe that some hospitals have already made the experiment, and we should welcome any news from institutions which have tried oil fuel. The proposal has been discussed more than once. Has it been tried and found successful ?

### BANK STAFFS AND HOSPITALS.

Too little is known of the quiet progress made by the Bankmen's appeal on behalf of the London Hospitals. It is organising regular contributions of, say, one shilling a month, on salary days, from the staffs of the different banks and their branches. The contributors in each branch can decide on the hospital to benefit by their subscriptions. Mr. Cuthbert Vaux, of 10 Clement's Lane, is the chairman of the appeal. At the end of 1921, the first year, £1,000 was collected, which increased to £4,000 and £6,000 respectively in the two succeeding years. Much has yet to be done before every considerable branch in London has its roll of subscribers, but since hospitals and banks are in constant relation with each other, it should be easy for the hospitals to interest someone in the branch offices of the banks within their area, so that the scheme may be extended. The object this year is to raise £10,000, and as the scheme has great possibilities of growth we hope that hospitals and the banks will co-operate heartily.

